# Green Synthesis of Silver Nanoparticles Using Spent Coffee Ground Extracts: Process Modelling and Optimization

**DOI:** 10.3390/nano12152597

**Published:** 2022-07-28

**Authors:** Antonio Zuorro, Annalaura Iannone, Selenia Miglietta, Roberto Lavecchia

**Affiliations:** 1 Department of Chemical Engineering, Materials and Environment, Sapienza University, 00184 Rome, Italy; annalaura.iannone@uniroma1.it (A.I.); roberto.lavecchia@uniroma1.it (R.L.); 2 Department of Human Anatomy, Histology, Forensic Medicine and Orthopedics, Sapienza University, 00185 Rome, Italy; selenia.miglietta@uniroma1.it

**Keywords:** green synthesis, silver nanoparticles, spent coffee grounds, agro-industrial wastes

## Abstract

Large amounts of spent coffee grounds (SCGs) are produced annually worldwide. SCGs contain high levels of phenolics and other bioactive compounds that make them a potential source of reducing and stabilizing agents for the synthesis of metal nanoparticles. This study investigates the use of SCG extracts as a green strategy to produce silver nanoparticles (AgNPs). SCG extracts were obtained using aqueous ethanol as the solvent and then contacted with a silver nitrate solution under the selected conditions. A central composite design coupled with response surface methodology was used to evaluate the effects of solvent composition (C = 30–70% *v*/*v*), silver-to-phenolic ratio (R = 3–7 mol/mol), temperature (T = 25–55 °C) and pH (10–12) on the production of AgNPs. Characterization of AgNPs by DLS, TEM and XRD techniques showed that they were highly crystalline with a narrow size distribution. Under optimal reaction conditions, AgNPs with an average size of about 10 nm and a zeta potential of −30.5 to −20.7 mV were obtained. Overall, the results of this study indicate that SCGs are a promising material for the green synthesis of small-sized and stable AgNPs.

## 1. Introduction

Silver nanoparticles (AgNPs) continue to attract attention from researchers due to their unique antibacterial, cytotoxic and catalytic properties, which make them ideal candidates for a variety of medical and non-medical applications [1,2,3]. This has stimulated interest in the development of new methods of synthesis in which toxic chemicals are replaced by natural compounds. Most of the proposed methods are based on the use of bioactive compounds from plants [4], with a special recent focus on the possibility of combining the principles of green chemistry with those of circular economy [5]. In this regard, the synthesis of AgNPs using agro-industrial or food wastes as a source of reducing and stabilizing agents could represent a valuable opportunity. Examples of this approach are the production of AgNPs using extracts from rice husks [6], bilberry and red currant wastes [7], tamarind shells [8] and mandarin peels [9].

Plant-derived materials contain several bioactive compounds, such as terpenoids, polyphenols, alkaloids, sugars, phenolic acids and proteins, that can reduce metal ions, allowing the formation of nanoparticles [10]. Some of these compounds also behave as capping agents, binding to the surface of nanoparticles and controlling their size and shape [11]. The degree of stabilization depends on the molecular features of the capping agent, such as the type, number and relative position of the functional groups interacting with the nanoparticles [12,13]. The synthesis of metal nanoparticles using plant materials has several advantages over traditional chemical and physical methods, including, among others, the absence of toxic compounds, the simplicity of the production process and the use of mild operating conditions [4,10,14]. Furthermore, studies on AgNPs suggest that the green synthesized nanoparticles have excellent antibacterial and antibiofilm activities, even higher than those of their commercial counterparts [15,16].

Coffee is one of the most popular beverages in the world and the second largest traded commodity after petroleum [17]. From the production of coffee beverages or the manufacturing of instant coffee, a solid residue known as spent coffee grounds (SCGs) is generated. According to recent estimates, about 15 million tons of SCGs are produced yearly [18]. Several strategies have been proposed to mitigate the problems associated with the management of this waste, including the production of biofuels, its transformation into activated carbon or the recovery of value-added components such as carbohydrates, proteins and polyphenols [19,20,21].

The presence of phenolic compounds and other substances with metal-reducing ability make SCGs potentially suitable for the production of nanoparticles. However, little attention has so far been given to the use of SCGs for this purpose. Baiocco et al. [22] showed that AgNPs with an average size of about 20 nm could be easily obtained at room temperature by contacting a silver nitrate solution with an ethanolic extract from SCGs. In another study, AgNPs–SCGs composites were prepared by directly adding SCGs to the AgNO_3_ solution [23]. AgNPs were formed on the surface of SCGs and provided the composites with antibacterial activity against *S. aureus* and *E. coli*. In yet another study, SCGs were previously hydrolyzed by treatment with HCl and then used to synthesize AgNPs [24]. The resulting nanoparticles had an average size of about 20 nm and exhibited antimicrobial activity against some Gram-negative bacteria.

Although limited in number, the above reports suggest that SCGs may represent a promising source of reducing and stabilizing agents for the green synthesis of AgNPs. Accordingly, the present study was undertaken to provide further support for the use of SCGs for this purpose. More specifically, we wanted to investigate in a quantitative manner the effects of the main process variables on the production of AgNPs from SCG extracts. A second objective was to determine whether it was possible to optimize the overall process and characterize the nanoparticles obtained under these conditions. To this end, a rigorous approach based on the design of experiments and the response surface methodology was used. The results obtained indicated that small-sized and stable AgNPs can be obtained by proper selection of the synthesis conditions.

## 2. Materials and Methods

### 2.1. Chemicals

Silver nitrate was purchased from Caelo (Hilden, Germany). Ethanol, sodium hydroxide and sodium carbonate were obtained from Carlo Erba (Milano, Italy). Hydrochloric acid (37 wt%), gallic acid (3,4,5-trihydroxybenzoic acid) and the Folin–Ciocalteu reagent were from Sigma-Aldrich Co. (St. Louis, MO, USA). 

All chemicals were used as received without further purification. Aqueous solutions were prepared using ultrapure water (18.2 MΩ cm). 

### 2.2. SCGs

SCGs were collected from several coffee bars in Rome (Italy). After removal of extraneous material, the waste was dried at 40 °C for 12–15 h in a forced-air dehydrator (Stöckli, Switzerland), thoroughly mixed and stored in the dark at room temperature until use.

### 2.3. Production of SCG Extracts

SCG extracts were obtained as described in [25]. Briefly, known amounts of SCGs and the solvent were loaded into thermostated (at ±0.1 °C) and magnetically stirred glass flasks. The extraction was carried out at 40 °C under continuous stirring for 3 h. Aqueous ethanol at different concentrations (10–90% *v*/*v*) was used as the solvent, while the liquid-to-solid ratio was kept at 10 mL/g. At the end of the extraction, the suspension was centrifuged at 7000× *g* for 10 min and the aqueous extract was recovered.

### 2.4. Synthesis of AgNPs

AgNPs were synthesized in 20-mL glass vials thermostated at the selected temperature and magnetically stirred at 400 rpm. The vials were loaded with appropriate amounts of 0.1 M silver nitrate solution and SCG extract. The pH of the reaction solution was adjusted to the desired value by addition of NaOH. The formation of AgNPs was monitored spectrophotometrically by measuring the intensity of the surface plasmon resonance (SPR) band of silver at about 430 nm. All experiments were made at least in duplicate. The reaction temperature was varied between 10 and 70 °C, the pH between 9 and 13, and the silver-to-phenolic ratio, defined as the ratio of Ag^+^ concentration to total phenolics, between 1 and 9 mol/mol.

### 2.5. Analytical Methods

Total phenolics were determined by the Folin–Ciocalteu method according to the procedure reported in [26]. The results were expressed as gallic acid equivalents (GAE) using a calibration curve obtained with gallic acid standards.

Spectrophotometric measurements were made with a double-beam UV-Vis spectrophotometer (mod. UV-2700, Shimadzu, Kyoto, Japan).

An X’Pert PRO diffractometer (Philips, Eindhoven, The Netherlands) was used for XRD measurements. The instrument was operated at 40 kV and 30 mA with Cu Kα radiation (λ = 1.5406 Å). The 2θ angle was varied from 20° to 80°. The step size was 0.04° and the counting time was 20 s per step. 

Nanoparticle size and zeta potential were measured using a Litesizer™ 500 instrument (Anton Paar, Graz, Austria).

Transmission electron microscopy (TEM) images were obtained with a Zeiss EM10 instrument (Carl Zeiss, Thornwood, NY, USA) operated at 60 kV. Samples were prepared by placing a drop of the nanoparticle solution onto a standard carbon-coated copper grid. Images were analyzed by the ImageJ software (ImageJ, National Institutes of Health, Bethesda, MD, USA).

### 2.6. Experimental Design

An experimental approach based on a Central Composite Design (CCD) [27] was used to investigate the effects of solvent composition (C), namely, the volume percentage of ethanol in the solvent, silver-to-phenolic ratio (R), temperature (T) and pH on the production of AgNPs. In all experiments, the reaction time was fixed to 5 h. The CCD consisted of a full two-level factorial design (2^4^ points), eight axial points at a distance ±α from the central point and six replicates of the central point. The value of α was taken as (2^4^)^1/4^ = 2 to ensure the orthogonality and rotatability of the design.

The variation range of each factor was determined based on the results of preliminary experiments and on previous studies. Then, the factor levels to be explored were evaluated. They are reported in Table 1 in actual and coded values. According to the DOE (Design of Experiments) terminology, actual levels denote the values of factors in dimensional units, while coded levels indicate their corresponding dimensionless values. Actual (*X_i_*) and coded (*x_i_*) levels are linked by the following relation:

(1) $$x_{i}=\frac{X_{i}-X_{i,0}}{\Delta X_{i}},$$

where *X_i_*_,0_ is the value of the *i*-th factor at the center-point level and Δ*X_i_* is the step change value for that factor.

The intensity of the SPR band at λ_max_ was taken as the response variable. Overall, the CCD consisted of 30 runs (Table 2), which were performed in random order to minimize the effects of uncontrolled factors.

The statistical analysis of the results was performed using the Design-Expert^®^ software (version 7.0.0, Stat-Ease, Inc., Minneapolis, MN, USA).

## 3. Results

### 3.1. Production of SCG Extracts

The moisture content of SCGs was 60.5 ± 1.8 wt% and was reduced to 6.4 ± 0.1 wt% after drying. The phenolic content was 30.25 ± 0.43 mg GAE/g, a value that is in line with those reported in previous studies [21,22,25,28], confirming that SCGs are an important source of phenolic compounds.

**Figure 1:**
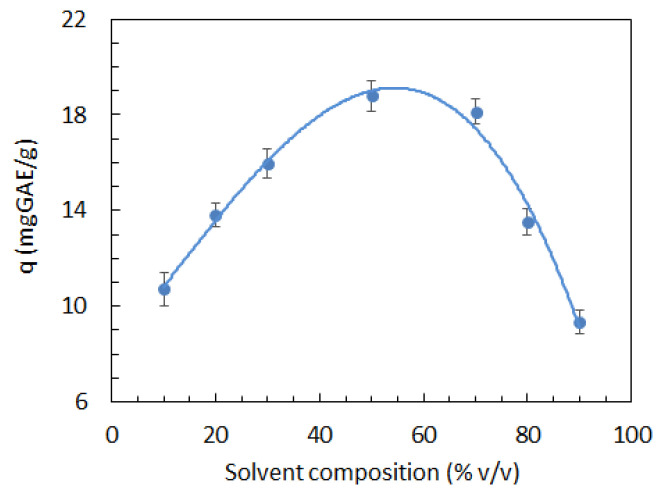
Effect of solvent composition on the amount of extracted phenolic compounds per unit dry weight of SCGs (*q*).

Preliminary experiments were made to investigate the effects of solvent composition on the extraction of phenolic compounds. The results shown in Figure 1 indicate that the highest extraction yields were achieved for an ethanol concentration in the solvent close to 50% *v*/*v*. However, since SCGs contain a variety of phenolic compounds with different Ag^+^ reducing abilities, the solvent composition that maximizes the overall extraction efficiency can differ from that for the optimal production of AgNPs. In addition, co-extracted non-phenolic compounds from SCGs could also contribute to the formation and/or stabilization of AgNPs. For this reason, solvent composition was included in the list of factors and its central point value was set to 50% *v*/*v*.

### 3.2. Spectrophotometric Characterization of AgNPs

**Figure 2:**
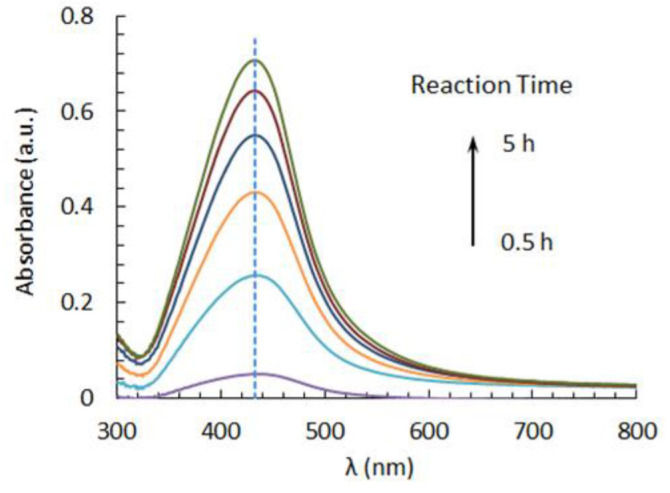
Evolution of the SPR band of AgNPs at 40 °C and pH 11 in reaction media containing silver nitrate and SCG extracts.

In Figure 2, some UV-Vis spectra of the solution containing silver nitrate and SCG extracts at different reaction times are displayed. The spectrum was dominated by a strong SPR band at about 430 nm, indicating the formation of AgNPs. The reaction was accompanied by a color change of the solution from pale yellow to deep brown and was completed within about 5 h. As is known, the intensity and sharpness of the SPR band can be related to the amount of nanoparticles produced and their size distribution, respectively. In particular, the sharper the peak, the narrower the size distribution [29].

### 3.3. Model Fitting 

Different mathematical models, including the linear, the two-factor interaction, the quadratic and the cubic models, were tested for their ability to fit the experimental design data. The best results were obtained with the quadratic model:

(2) $$y=a_{0}+\sum_{i}a_{i}x_{i}+\sum_{i}a_{ii}x_{i}^{2}+\sum_{i}\sum_{j}a_{ij}x_{i}x_{j},$$

where *y* is the intensity of the SPR band at *λ_max_* and *x_i_* are the coded independent variables. In addition to the intercept (*a*_0_), Equation (2) contains 4 linear (*a_i_*), 4 quadratic (*a_ii_*), and 6 interaction (*a_ij_*) coefficients, for a total of 15 parameters. The full quadratic model was reduced by stepwise regression to keep only the statistically significant terms, while preserving the hierarchy of higher order terms. By this procedure, the following reduced model was obtained:

(3) $$y=a_{0}+a_{1}x_{1}+a_{2}x_{2}+a_{3}x_{3}+a_{4}x_{4}+a_{11}x_{1}^{2}+a_{44}x_{4}^{2}+a_{23}x_{2}x_{3}+a_{24}x_{2}x_{4},$$

**Figure 3:**
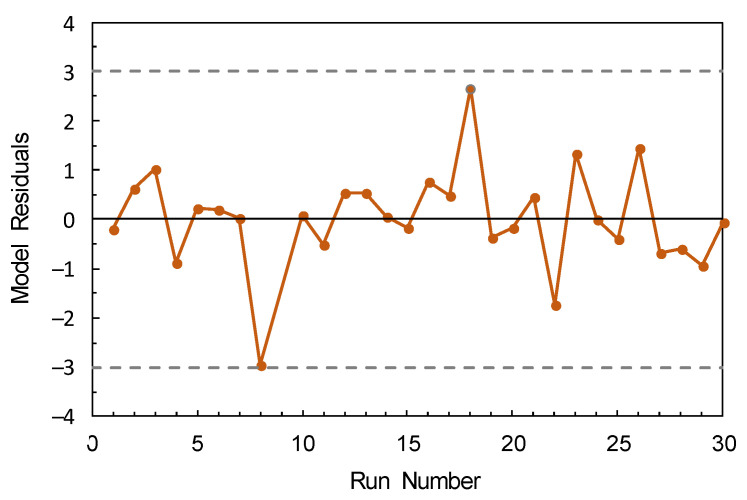
Model residuals as a function of run number.

The nine parameters were estimated by the least-square methods, giving the results listed in Table 3. The *R*-squared and adjusted *R*-squared values were 0.9637 and 0.9499, respectively. As can be seen from the ANOVA results shown in Table 4, the model was statistically significant (*p* < 0.0001) and that the lack-of-fit was not significant (*p* = 0.1972). Studentized model residuals were randomly scattered between –3 and +3 (Figure 3), indicating that the ANOVA assumptions were met.

### 3.4. Analysis of Influencing Factors 

**Figure 4:**
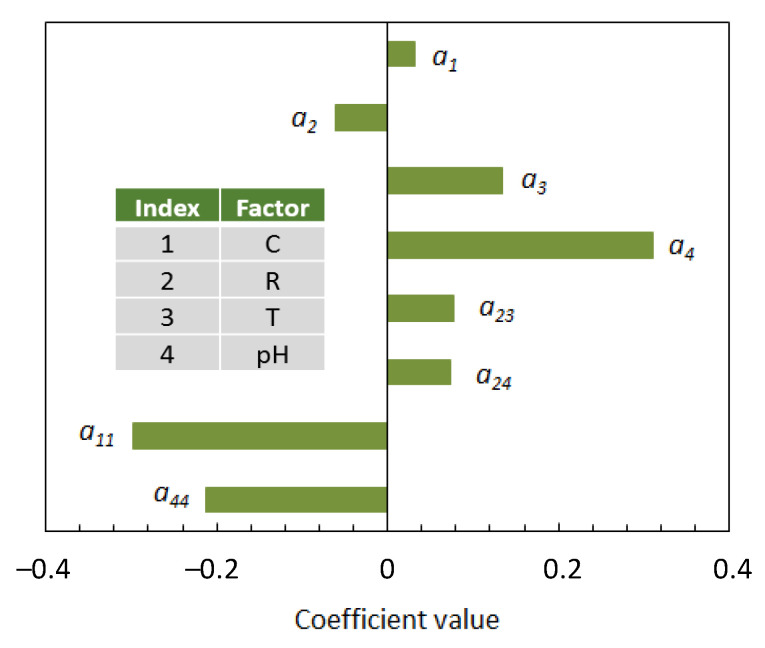
Pareto chart for the model coefficients.

Figure 4 shows the Pareto chart for the model coefficients. From this diagram, the following points can be made:

(a) Two of the four investigated factors, namely, the ethanol concentration in the aqueous solvent (C) and the pH of the reaction medium, affected the production of AgNPs through both a linear and a quadratic term;
(b) Concerning the linear terms, the silver-to-phenolic ratio (R) gave a negative contribution on the response variable, while the contributions of the remaining factors were positive and increased in in the order: C < T < pH;
(c) There were two positive interactions: between the silver-to-phenolic ratio (R) and the temperature (T), and between the silver-to-phenolic ratio (R) and the pH, indicating that the R factor had a more pronounced effect on the production of AgNPs at higher temperature and pH.

For a better appreciation of the effects of the investigated factors on the production of AgNPs, an analysis of perturbation and response surface plots was performed.

**Figure 5:**
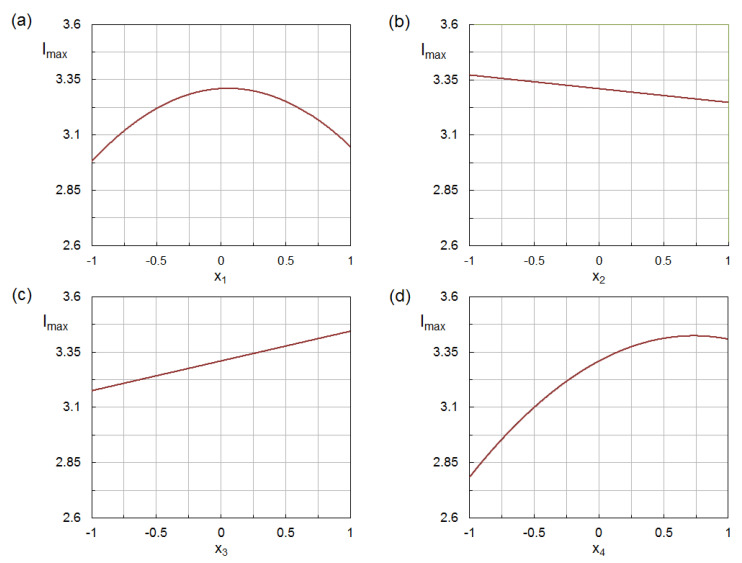
Perturbation plots for the four factors: (**a**) solvent composition; (**b**) silver-to-phenolic ratio; (**c**) temperature; and (**d**) pH. Each diagram was plotted by keeping the levels of the other three factors at their central value.

Perturbation plots allow a comparison of the individual effects of factors on the response variable at a specific point in the design space. Each factor was changed over its full factorial range (−1, 1), while setting the other factors to their central point values (0). As is evident from Figure 5, the response variable varied non-monotonically with solvent composition and pH, which is consistent with the presence of quadratic terms for these factors in the model equation. Concerning the silver-to-phenolic ratio and the temperature, a negative linear dependence and a positive linear dependence were, respectively, observed. From the slopes of these lines, the higher sensitivity of the response variable to temperature can also be deduced.

**Figure 6:**
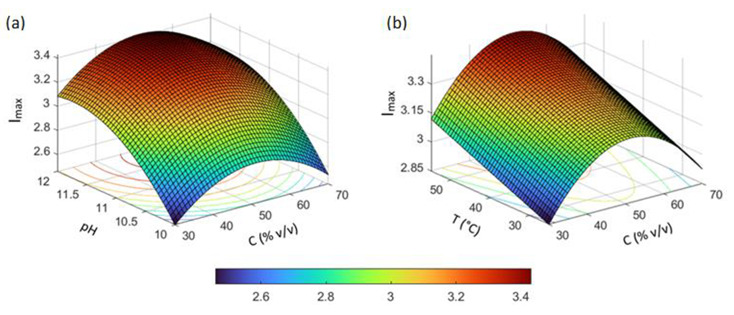
Representative response surface plots showing the effects on the intensity of the SPR band (*I*_max_) of: (**a**) pH and solvent composition (C); (**b**) temperature (T) and solvent composition (C). For each plot, the levels of the other factors were fix at their central values (C = 50% *v*/*v*; R = 5 mol/mol; T = 40 °C; pH = 11).

**Figure 7:**
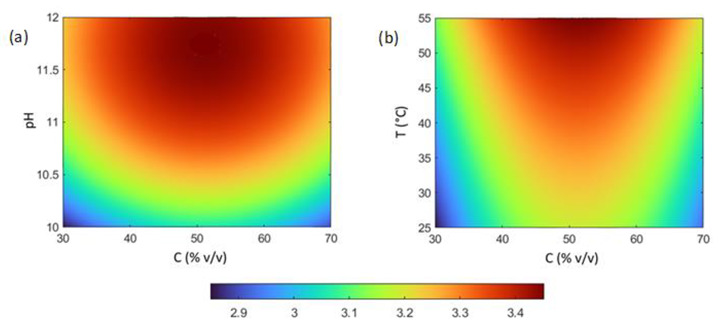
Representative contour plots showing the effects on the intensity of the SPR band (*I*_max_) of: (**a**) pH and solvent composition (C); (**b**) temperature (T) and solvent composition (C). For each plot, the levels of the other factors were fixed at their central values (C = 50% *v*/*v*; R = 5 mol/mol; T = 40 °C; pH = 11).

Response surface and contour plots were generated to visualize the combined effects of influencing factors. Some representative plots, obtained by setting two of the four factors constant at their central point values, are shown in Figure 6 and Figure 7. The curvature of the response surfaces reflects the quadratic effects of factors and their shape also provides an indication of the interaction between factors. 

### 3.5. Process Optimization 

An examination of the model structure (Equation (2)) and its 3D and 2D graphical representations (Figure 6 and Figure 7) clearly indicate that the production of AgNPs can be optimized by appropriate selection of process conditions. The search for the optimum was performed over the whole investigated domain (–α ≤ *x_i_* ≤ α) by maximizing the response variable. For this purpose, the gradient descent method was used with different randomly selected starting point. The following result was obtained: C = 49.2% *v*/*v*; R = 8 mol/mol; T = 65 °C; pH = 12.2. The predicted response was 3.836, with a 95% confidence interval of 3.620–4.053. The model was validated by performing a new experiment under these optimum conditions, which gave: *y_exp_* = 3.909 ± 0.08. This value differs by about 1.8% from the predicted one and falls within the 95% confidence interval, further supporting the reliability of the developed model.

### 3.6. Characterization of AgNPs

**Figure 8:**
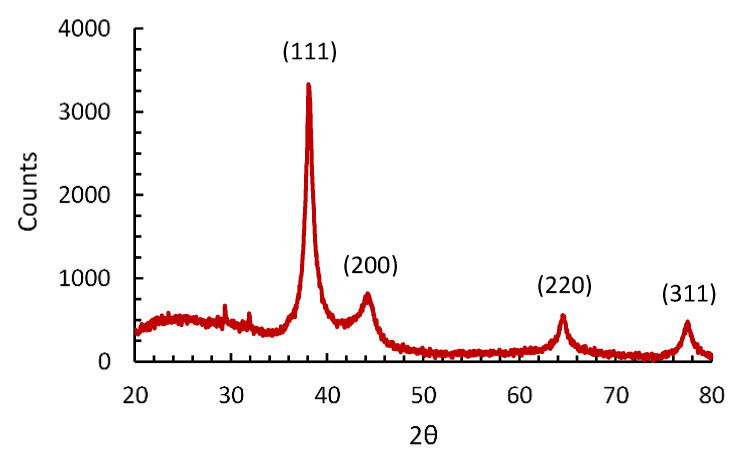
XRD pattern of the synthesized AgNPs.

Figure 8 shows the XRD pattern of the synthesized AgNPs. In the 2θ range of 20–80°, there were four sharp peaks at 38.1°, 44.2°, 64.5° and 77.5°, corresponding, respectively, to the planes (111), (200), (220) and (311) of the FCC structure of silver. The peak at 38.1° was the most intense, indicating that the preferred orientation was along the (111) plane.

The average nanoparticle size obtained from dynamic light scattering (DLS) measurements ranged from about 23.7 to 39.9 nm, depending on the reaction conditions, while the zeta potential varied between −30.5 and −20.7 mV. 

**Figure 9:**
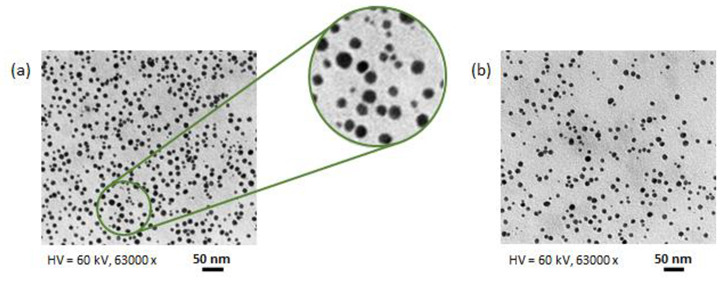
TEM images of the synthesized AgNPs under (**a**) optimal (C = 49.2% *v*/*v*; R = 8 mol/mol; T = 65 °C; pH = 12.2) and (**b**) suboptimal (C = 50% *v*/*v*; R = 5 mol/mol; T = 40 °C; pH = 11) process conditions.

Some TEM images of the produced nanoparticles are displayed in Figure 9. These images reveal that the AgNPs were spherical. The average nanoparticle size derived from the analysis of TEM images ranged from about 8.2 to 10.4 nm. These values are smaller than those from DLS, which can be attributed to the fact that DLS measures the hydrodynamic diameter of nanoparticles rather than the physical one [30]. Furthermore, the capping layer around the nanoparticles is transparent to electrons and therefore does not contribute to the size obtained from TEM measurements [31]. 

## 4. Discussion

This study was designed to investigate the use of SCGs as a source of reducing and stabilizing agents for the synthesis of AgNPs. The results obtained indicate that SCGs are a suitable material for this purpose and that the production process can be optimized by operating at 65 °C and pH 12.2, using aqueous ethanol at 49.2% as the extraction solvent and a silver-to-phenolic ratio of 8 mol/mol.

To provide an interpretation of these results, the characteristics of SCG extracts should first be considered. SCGs are the solid residue left after brewing of coffee beans. The latter are obtained by subjecting the seeds of coffee cherries, known as green coffee beans, to roasting. Green coffee is mainly composed of carbohydrates (55–65.5%), lipids (10–18%), *N*-containing compounds (11–15%), purine alkaloids (0.8–4%), chlorogenic acids (6.7–9.2%) and minerals (3–5.4%) [32]. In addition to chlorogenic acids, other bioactive compounds, such as diterpenes, caffeine and trigonelline, are also present [33]. When the beans are roasted, the characteristic organoleptic properties of coffee are developed. The variables that most affect the quality of the final product are the treatment temperature (typically, 200–260 °C) and duration (typically, 5–15 min) [34]. During roasting, a series of chemical reactions occur, like pyrolysis and the Maillard reaction, that modify the composition of coffee beans [35,36]. In particular, some chlorogenic acids and other coffee components are degraded while new volatile and non-volatile compounds are formed.

SCGs contain several compounds with reducing activity, such as chlorogenic, gallic, caffeic, ellagic, ferulic, *p*-coumaric and *p*-hydroxybenzoic acids, esters of caffeic and ferulic acids with quinic acid, caffeine, rutin, quercetin and trigonelline [37]. In this study, ethanol–water mixtures at different ratios were used to recover bioactive compounds from SCGs. As is known, in a solid–liquid extraction process, the properties of the solvent strongly affect the overall extraction yield as well as the relative amounts of extracted compounds [38]. The results presented here showed that changes in the solvent composition affected both the production of AgNPs and the amount of phenolic compounds extracted from SCGs. Furthermore, the optimal solvent composition for nanoparticle production (C = 49.2% *v*/*v*) was very close to that of the maximum yield of phenolics extraction, which suggests that phenolic compounds are the main bioactive components involved in the synthesis of AgNPs. 

Regarding the mechanism of nanoparticle formation, it should be considered that the reducing ability of phenolic compounds is related to the presence in their molecules of one or more hydroxyl groups [6,39]. In the case of AgNPs, silver ions are reduced to metals by transfer of electrons provided by the phenolic compounds [40]. 

For the generic phenolic compound (*PC*), the following electron transfer process, involving the oxidation of *PC* to its quinone form (*PCQ*) and the reduction of silver ions to metallic silver, can be assumed:

(4) $$PC\to PCQ+n\;H^{+}+n\;e^{-},$$

(5) $$n\;Ag^{+}+n\;e^{-}\to n\;Ag^{0},$$

**Figure 10:**
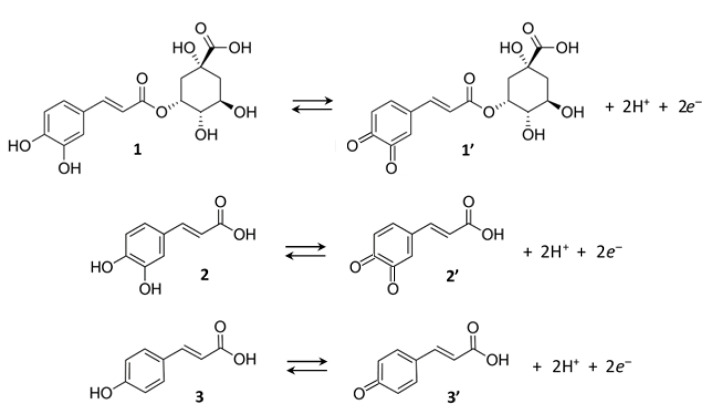
Oxidation of chlorogenic acid (1), caffeic acid (2) and *p*-coumaric acid (3) to their corresponding quinone forms (1′, 2′ and 3′).

Figure 10 shows the oxidation reactions for three of the most common phenolic acids in SCGs (chlorogenic acid, caffeic acid and *p*-coumaric acid).

Combining reactions (4) and (5) gives the following overall reaction:

(6) $$PC+n\;Ag^{+}\to PCQ+n\;H^{+}+n\;Ag^{0}.$$

According to the classical nucleation theory [41], the produced silver atoms (*Ag*^0^) can aggregate to form metal clusters (or embryonic nuclei):

(7) $$m\;Ag^{0}\to {\left(Ag^{0}\right)}_{m}.$$

Nuclei with a radius smaller than the critical one (*r* < r*) will dissolve in the solution, while those of greater radius (*r* ≥ r*) will grow up to form AgNPs. Capping agents can interact with the surface of nanoparticles and form a coating layer which prevents aggregation affecting, at the same time, the final size of the nanoparticles. In the case of phenolic acids, the build-up of this layer is determined by the binding of their carboxylic moieties to the nanoparticle surface followed by the formation of intermolecular hydrogen bonds between hydroxyl groups of surface-capped molecules [39]. However, other functional groups of phenolic compounds could also play a role in stabilization. This is the case, for example, of catechol groups of some phenolic acids, which can easily adsorb on metal surfaces [42].

The positive effect of increasing pH on the production of AgNPs can be explained by the fact that H^+^ ions are released during the oxidation of phenolic compounds (Equation (4)), which means that the nucleation of nanoparticles is favored under alkaline conditions. Nevertheless, at high pH, the production of Ag_2_O can also occur [43]:

(8) $$2\;Ag+2\;OH^{-}\to Ag_{2}O+H_{2}O,$$

together with a weakening of the interactions of surface-capped molecules with the nanoparticle surface [39]. Both phenomena could lead to a progressive reduction in the positive effect of pH and be responsible for the occurrence of an optimum pH (in this case, pH 12.2) for the synthesis of AgNPs.

The analysis conducted also revealed a positive influence of temperature on the synthesis of AgNPs, which can be primarily related to its positive effect on the reaction kinetics. However, due to the complexity of the reaction system and the presence in the reaction medium of many different compounds with reducing and/or stabilizing abilities, it is difficult to provide an adequate explanation to the observed behavior. The detected interaction between temperature and silver-to-phenolic ratio could be a reflection of this complexity. For example, temperature has a different impact on the reduction of silver ions by phenolic compounds and the formation of the coating layer to which these compounds also contribute [44,45]. Although the results of the present study do not allow us to identify their contributions, the methodology used can be adapted to include more response variables, such as the particle size and morphology, that could help elucidate the underlying mechanisms.

## 5. Conclusions

The results of this study indicate that small-sized and stable AgNPs can be obtained using SCGs as a source of reducing and stabilizing agents. The production process was investigated by a rigorous approach based on the response surface methodology, which provided the following set of optimal operating conditions: C = 49.2% *v*/*v*; R = 8 mol/mol; T = 65 °C and pH = 12.2.

The proposed process is very simple to perform and easily scalable. Furthermore, the use of aqueous ethanol as solvent and the exploitation of a plant waste make it environmentally friendly and compliant with the circular economy principles.

## Figures and Tables

**Table 1:** Actual and coded levels of factors for the CCD.

Factor	Factor Level					Unit
	−α	−1	0	+1	+α	
Solvent composition (C)	10	30	50	70	90	% *v*/*v*
Silver-to-phenolic ratio (R)	1	3	5	7	9	mol/mol
Temperature (T)	10	25	40	55	70	°C
pH	9	10	11	12	13	−

**Table 2:** Experimental design layout and observed response (*y*). SO and RO are the standard and the run order of experiments.

SO	RO	Factor Level				*y*
		*x*_1_	*x*_2_	*x*_3_	*x*_4_	
1	22	−1	−1	−1	−1	2.380
2	26	+1	−1	−1	−1	2.733
3	3	−1	+1	−1	−1	2.201
4	29	+1	+1	−1	−1	2.087
5	7	−1	−1	+1	−1	2.653
6	24	+1	−1	+1	−1	2.714
7	18	−1	+1	+1	−1	2.775
8	8	+1	+1	+1	−1	2.329
9	14	−1	−1	−1	+1	3.015
10	20	+1	−1	−1	+1	3.058
11	4	−1	+1	−1	+1	2.798
12	21	+1	+1	−1	+1	2.986
13	30	−1	−1	+1	+1	3.118
14	1	+1	−1	+1	+1	3.170
15	25	−1	+1	+1	+1	3.268
16	13	+1	+1	+1	+1	3.418
17	11	−2	0	0	0	2.012
18	16	+2	0	0	0	2.257
19	12	0	−2	0	0	3.484
29	17	0	+2	0	0	3.230
21	15	0	0	−2	0	3.025
22	19	0	0	+2	0	3.545
23	10	0	0	0	−2	1.846
24	6	0	0	0	+2	3.099
25	23	0	0	0	0	3.430
26	2	0	0	0	0	3.366
27	5	0	0	0	0	3.332
28	28	0	0	0	0	3.256
29	27	0	0	0	0	3.247
30	9	0	0	0	0	3.235

**Table 3:** Estimated coefficients for the reduced model described by Equation (2) with the associated standard errors (SE) and 95%-confidence intervals (CI).

Coefficient	Term	Value	SE	Low CI	High CI
*a*_0_	intercept	3.310	0.031	3.24	3.37
*a*_1_	C	0.032	0.022	−0.013	0.078
*a*_2_	R	−0.062	0.022	−0.11	−0.016
*a*_3_	T	0.134	0.022	0.089	0.18
*a*_4_	pH	0.311	0.022	0.27	0.36
*a*_11_	C × C	−0.297	0.020	−0.34	−0.25
*a*_44_	pH × pH	−0.212	0.020	−0.25	−0.17
*a*_23_	R × T	0.078	0.027	0.022	0.13
*a*_24_	R × pH	0.075	0.027	0.019	0.13

**Table 4:** ANOVA results for the reduced model described by Equation (2). DF denotes the degrees of freedom, SS the sum of squares, MS the mean squares, F the *F*-value and p the *p*-value.

Source	DF	SS	MS	*F*	*p*
Model	8	6.497	0.812	69.67	<0.0001
Residual error	21	0.245	0.012		
Lack-of-fit	16	0.214	0.013	2.19	0.1972
Pure error	5	0.031	0.006		
Total	29	6.742			

## Data Availability

Data is contained within the article.

## References

[1] Zahoor M., Nazir N., Iftikhar M., Naz S., Zekker I., Burlakovs J., Uddin F., Kamran A.W., Kallistova A., Pimenov N. (2021). A review on silver nanoparticles: Classification, various methods of synthesis, and their potential roles in biomedical applications and water treatment. Water.

[2] Yaqoob A.A., Umar K., Ibrahim M.N.M. (2020). Silver nanoparticles: Various methods of synthesis, size affecting factors and their potential applications—A review. Appl. Nanosci..

[3] Nasrollahzadeh M., Mahmoudi-Gom Yek S., Motahharifar N., Ghafori Gorab M. (2019). Recent developments in the plant-mediated green synthesis of Ag-based nanoparticles for environmental and catalytic applications. Chem. Rec..

[4] Shumail H., Khalid S., Ahmad I., Khan H., Amin S., Ullah B. (2021). A review on green synthesis of silver nanoparticles through plants. Endocr. Metab. Immun. Disord. Drug Targets.

[5] Omran B.A., Baek K.-H. (2021). Valorization of agro-industrial biowaste to green nanomaterials for wastewater treatment: Approaching green chemistry and circular economy principles. J. Environ. Manag..

[6] Liu Y.-S., Chang Y.-C., Chen H.-H. (2018). Silver nanoparticle biosynthesis by using phenolic acids in rice husk extract as reducing agents and dispersants. J. Food Drug Anal..

[7] Zuorro A., Iannone A., Natali S., Lavecchia R. (2019). Green synthesis of silver nanoparticles using bilberry and red currant waste extracts. Processes.

[8] Tade R.S., Nangare S.N., Patil P.O. (2020). Agro-industrial waste-mediated green synthesis of silver nanoparticles and evaluation of its antibacterial activity. Nano Biomed. Eng..

[9] Omran B.A., Aboelazayem O., Nassar H.N., El-Salamony R.A., El-Gendy N.S. (2021). Biovalorization of mandarin waste peels into silver nanoparticles and activated carbon. Int. J. Environ. Sci. Technol..

[10] Makarov V.V., Love A.J., Sinitsyna O.V., Makarova S.S., Taliansky M.E., Yaminsky I.V., Kalinina N.O. (2014). “Green” nanotechnologies: Synthesis of metal nanoparticles using plants. Acta Nat..

[11] Rauwel P., Küünal S., Ferdov S., Rauwel E. (2015). A review on the green synthesis of silver nanoparticles and their morphologies studied via TEM. Adv. Mat. Sci. Eng..

[12] Restrepo C.V., Villa C.C. (2021). Synthesis of silver nanoparticles, influence of capping agents, and dependence on size and shape: A review. Environ. Nanotechnol. Monit. Manag..

[13] Bhutto A.A., Kalay S., Sherazi S.T.H., Culha M. (2018). Quantitative structure–activity relationship between antioxidant capacity of phenolic compounds and the plasmonic properties of silver nanoparticles. Talanta.

[14] Rozhin A., Batasheva S., Kruychkova M., Cherednichenko Y., Rozhina E., Fakhrullin R. (2021). Biogenic silver nanoparticles: Synthesis and application as antibacterial and antifungal agents. Micromachines.

[15] Salayová A., Bedlovičová Z., Daneu N., Baláž M., Lukáčová Bujňáková Z., Balážová Ľ., Tkáčiková Ľ. (2021). Green synthesis of silver nanoparticles with antibacterial activity using various medicinal plant extracts: Morphology and antibacterial efficacy. Nanomaterials.

[16] Barabadi H., Mojab F., Vahidi H., Marashi B., Talank N., Hosseini O., Saravanan M. (2021). Green synthesis, characterization, antibacterial and biofilm inhibitory activity of silver nanoparticles compared to commercial silver nanoparticles. Inorg. Chem. Commun..

[17] Campos-Vega R., Loarca-Pina G., Vergara-Castaneda H.A., Oomahb B.D. (2015). Spent coffee grounds: A review on current research and future prospects. Trends Food Sci. Technol..

[18] Kamil M., Ramadan K.M., Awad O.I., Ibrahim T.K., Inayat A., Ma X. (2019). Environmental impacts of biodiesel production from waste spent coffee grounds and its implementation in a compression ignition engine. Sci. Total Environ..

[19] Murthy P.S., Madhava Naidu M. (2012). Sustainable management of coffee industry by-products and value addition—A review. Resour. Conserv. Recycl..

[20] Massaya J., Prates Pereira A., Mills-Lamptey B., Benjamin J., Chuck C.J. (2019). Conceptualization of a spent coffee grounds biorefinery: A review of existing valorisation approaches. Food Bioprod. Process..

[21] McNutt J., He Q.S. (2019). Spent coffee grounds: A review on current utilization. J. Ind. Eng. Chem..

[22] Baiocco D., Lavecchia R., Natali S., Zuorro A. (2016). Production of metal nanoparticles by agro-industrial wastes: A green opportunity for nanotechnology. Chem. Eng. Trans..

[23] Chien H.-W., Kuo C.-J., Kao L.-H., Lin G.-Y., Chen P.-Y. (2019). Polysaccharidic spent coffee grounds for silver nanoparticle immobilization as a green and highly efficient biocide. Int. J. Biol. Macromol..

[24] Panzella L., Cerruti P., Aprea P., Paolillo R., Pellegrino G., Moccia F., Condorelli G.G., Vollaro A., Ambrogi V., Catania M.R. (2020). Silver nanoparticles on hydrolyzed spent coffee grounds (HSCG) for green antibacterial devices. J. Clean. Prod..

[25] Zuorro A., Lavecchia R. (2011). Polyphenols and energy recovery from spent coffee grounds. Chem. Eng. Trans..

[26] Zuorro A., Iannone A., Lavecchia R. (2019). Water-organic solvent extraction of phenolic antioxidants from brewers’ spent grain. Processes.

[27] Montgomery D.C. (2017). Design and Analysis of Experiments.

[28] Zuorro A. (2015). Optimization of polyphenol recovery from espresso coffee residues using factorial design and response surface methodology. Sep. Purif. Technol..

[29] Nizamov T.R., Evstaf’Ev I.V., Olenin A.Y., Lisichkin G.V. (2014). The formation of mono- and bimetallic silver-containing seed nanoparticles. Colloid J..

[30] Cumberland S., Lead J. (2009). Particle size distributions of silver nanoparticles at environmentally relevant conditions. J. Chromatogr. A.

[31] Diegoli S., Manciulea A.L., Begum S., Jones I.P., Lead J.R., Preece J.A. (2008). Interaction between manufactured gold nanoparticles and naturally occurring organic macromolecules. Sci. Total Environ..

[32] Brzezicha J., Błażejewicz D., Brzezińska J., Grembecka M. (2021). Green coffee VS dietary supplements: A comparative analysis of bioactive compounds and antioxidant activity. Food Chem. Toxicol..

[33] Jeszka-Skowron M., Sentkowska A., Pyrzyńska K., De Peña M.P. (2012). Chlorogenic acids, caffeine content and antioxidant properties of green coffee extracts: Influence of green coffee bean preparation. Eur. Food Res. Technol..

[34] Getaneh E., Fanta S.W., Satheesh N. (2020). Effect of broken coffee beans particle size, roasting temperature, and roasting time on quality of coffee beverage. J. Food Qual..

[35] Muñoz A.E., Hernández S.S., Tolosa A.R., Burillo S.P., Olalla Herrera M. (2020). Evaluation of differences in the antioxidant capacity and phenolic compounds of green and roasted coffee and their relationship with sensory properties. LWT-Food Sci. Technol..

[36] Moreira A.S.P., Nunes F.M., Domingues M.R., Coimbra M.A. (2012). Coffee melanoidins: Structures, mechanisms of formation and potential health impacts. Food Funct..

[37] Kovalcik A., Obruca S., Marova I. (2018). Valorization of spent coffee grounds: A review. Food Bioprod. Process..

[38] Zuorro A., Maffei G., Lavecchia R. (2014). Effect of solvent type and extraction conditions on the recovery of phenolic compounds from artichoke waste. Chem. Eng. Trans..

[39] Yoosaf K., Ipe B.I., Suresh C.H., Thomas K.G. (2007). In situ synthesis of metal nanoparticles and selective naked-eye detection of lead ions from aqueous media. J. Phys. Chem. C.

[40] Laguerre M., Lecomte J., Villeneuve P. (2014). The physico-chemical basis of phenolic antioxidant activity. Lipid Technol..

[41] Lee J., Yang J., Kwon S.G., Hyeon T. (2016). Nonclassical nucleation and growth of inorganic nanoparticles. Nat. Rev. Mater..

[42] Amini S.M., Akbari A. (2019). Metal nanoparticles synthesis through natural phenolic acids. IET Nanobiotechnol..

[43] Adegboyega N.F., Sharma V.K., Siskova K., Zbořil R., Sohn M., Schultz B.J., Banerjee S. (2013). Interactions of aqueous Ag+ with fulvic acids: Mechanisms of silver nanoparticle formation and investigation of stability. Environ. Sci. Technol..

[44] Mashwani Z.-U.-R., Khan T., Khan M.A., Nadhman A. (2015). Synthesis in plants and plant extracts of silver nanoparticles with potent antimicrobial properties: Current status and future prospects. Appl. Microbiol. Biotechnol..

[45] Zuorro A., Iannone A., Lavecchia R., Natali S. (2021). Green synthesis of gold nanoparticles using kiwifruit juice. Chem. Eng. Trans..

